# Supramolecular Assemblies of Fluorescent Nitric Oxide Photoreleasers with Ultrasmall Cyclodextrin Nanogels

**DOI:** 10.3390/molecules28155665

**Published:** 2023-07-26

**Authors:** Tassia J. Martins, Cristina Parisi, Yota Suzuki, Takeshi Hashimoto, Antonia Nostro, Giovanna Ginestra, Takashi Hayashita, Salvatore Sortino

**Affiliations:** 1 Laboratory of Photochemistry, Department of Drug and Health Sciences, University of Catania, I-95125 Catania, Italy; tassia.joimartins@unict.it (T.J.M.); cristina.parisi@unict.it (C.P.); 2 Department of Materials and Life Sciences, Faculty of Science and Technology, Sophia University, Tokyo 102-8554, Japan; 3 Graduate School of Science and Engineering, Saitama University, Saitama 338-8570, Japan; 4 Department of Chemical, Biological, Pharmaceutical and Environmental Sciences, University of Messina, V.le, F. Stagno d’Alcontres, 31, I-98166 Messina, Italygginestra@unime.it (G.G.)

**Keywords:** light, nitric oxide, cyclodextrins, gels

## Abstract

Developing biocompatible nitric oxide (NO) photoreleasing nanoconstucts is of great interest in view of the large variety of biological roles that NO plays and the unique advantage light offers in controlling NO release in space and time. In this contribution, we report the supramolecular assemblies of two NO photodonors (NOPDs), NBF-NO and RHD-NO, as water-dispersible nanogels, ca. 10 nm in diameter, based on γ-cyclodextrins (γ-CDng). These NOPDs, containing amino-nitro-benzofurazan and rhodamine chromophores as light harvesting antennae, can be activated by visible light, are highly hydrophobic and can be effectively entrapped within the γ-CDng. Despite being confined in a very restricted environment, neither NOPD suffer self-aggregation and preserve their photochemical and photophysical properties well. The blue light excitation of the weakly fluorescent γ-CDng/NBF-NO complex results in effective NO release and the concomitant generation of the highly green, fluorescent co-product, which acts as an optical NO reporter. Moreover, the green light excitation of the persistent red fluorescent γ-CDng/RHD-NO triggers NO photorelease without significantly modifying the emission properties. The activatable and persistent fluorescence emissions of the NOPDs are useful for monitoring their interactions with the Gram-positive methicillin-resistant *Staphylococcus aureus*, whose growth is significantly inhibited by γ-CDng/RHD-NO upon green light irradiation.

## 1. Introduction

Nitric oxide (NO) is a key mediator of many physiological processes, including vasodilation, neurotransmission and hormone secretion [1]. Moreover, the last two decades have witnessed additional and important roles this inorganic free radical plays, especially in cancer [2], wound-healing [3] and bacterial infections [4]. NO shows attractive advantages over conventional chemotherapy drugs and antibiotics, including the absence of multidrug resistance, multitarget activity, a broad spectrum of action and, as a result of its ephemeral nature, the confinement of its cytotoxic action over short distances (<200 µm) from its generation site. This has opened intriguing horizons for potential and innovative, therapeutic approaches based on the use of NO as unconventional therapeutic as either an alternative to or in combination with conventional drugs.

Due to its gaseous nature, it is difficult to deliver NO. This issue has inspired the development of a range of NO-releasing precursors that are able to store NO as a part of their molecular or macromolecular skeletons and deliver it in a controlled manner [5,6,7,8,9]. The spatiotemporal control of NO release is of particular relevance, since the therapeutic effects are strictly dependent on its doses and site of action. This has made NO releasers activatable by light stimuli, usually named NO photodonors (NOPDs), which are very appealing in view of the high spatiotemporal precision in the delivery process that they offer, in comparison to spontaneous NO releasers [10,11,12,13,14,15,16].

For practical bio-applications, the solubility of NOPDs in aqueous medium is highly desirable. Nanotechnology is very helpful in this regard, offering a variety of water-dispersible nanocontainers suitable for the covalent and non-covalent integration of otherwise water-insoluble NOPDs [17]. Nanotechnogical approaches also allow the concentration of a large number of NOPDs at a very restricted volume, increasing light harvesting properties and enabling the production of a burst of NO in a very small area.

In contrast to spontaneous NO releasers, one key aspect to be considered in the case of NOPDs encapsulated in host nanocarriers is related to the preservation of their photochemical behavior within the host. This is not trivial since, in many cases, specific intra- and inter-molecular guests’ interactions and/or the presence of steric constrains can dramatically affect the efficiency or nature, or both, of the desired photochemical reaction (i.e., NO photorelease).

**Scheme 1:**
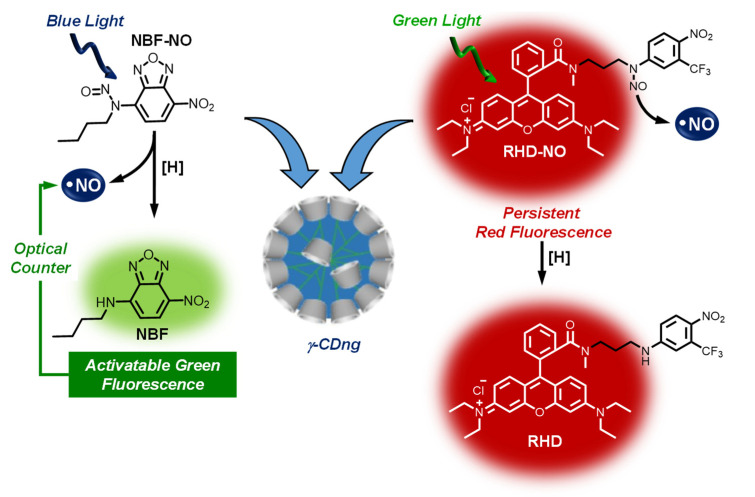
Sketch of the γ-CDng and molecular structures and working principles of the water-insoluble NOPDs.

Among the large variety of host nanocarriers, nanogels represent a class of promising and innovative materials as next-generation drug-delivery systems [18]. They are usually composed of polymeric chains dispersed in a large volume of liquid to form three-dimensional networks [19]. The main features of nanogels include relatively high drug encapsulation capacity, controllable dimensions and very low toxicity. Moreover, due to their small size and composition they show excellent optical transparency, which is an ideal pre-requisite for spectroscopic investigations and the photochemical activation of chromo-fluorogenic guest molecules. In recent years, cyclodextrins (CDs), cyclic oligosaccharides composed of six (α-CD), seven (β-CD) or eight (γ-CD) glucopyranose units linked by 1,4 bonds [20], have entered the limelight as suitable building blocks for the fabrication of nanogels [21,22,23,24]. The typical ability of CDs to form inclusion complexes with a variety of guest molecules due to differently sized hydrophobic cavities, is retained or even improved when CDs are polymerized into nanogels. Very recently, a novel method using emulsion polymerization and [dilauryl(dimethyl)]ammonium bromide for the preparation of uniform and ultrasmall γ-CD nanogels (γ-CDng) (Scheme 1) ca. 10 nm in diameter and with a zeta potential of ca. −1.5 mV at neutral pH has been reported [25]. This material exhibited superior inclusion properties than native CDs and, due to their reduced sizes, is expected to be able to avoid the undesired toxicity or tissue accumulation issues that are typical for larger sized nanogels [24].

On these grounds and stimulated by our ongoing interest on NOPDs for bio-applications [10,11,17], we considered of value of exploring the supramolecular assemblies of the above γ-CDng with two recently developed NOPDs, NBF-NO and RHD-NO (Scheme 1), based on amino-nitro-benzofurazan and rhodamine chromophores as light harvesting antennae, respectively [26,27]. The choice of these NOPDs is not random and is motivated by their common insolubility in water and their different photochemical properties. In fact: (i) NBF-NO is not fluorescent and its activation with blue light triggers the NO release with the concomitant generation of the highly green fluorescent product NBF, which acts as an optical reporter to monitor the NO release in real time [26]; (ii) RHD-NO exhibits persistent red fluorescence, and its activation with green light triggers the NO release without affecting the emissive properties [27]. In this contribution, we report the photochemical and photophysical properties of the above supramolecular assemblies and some preliminary antibacterial tests on methicillin-resistant *Staphylococcus aureus* (MRSA).

## 2. Results and Discussion

**Figure 1:**
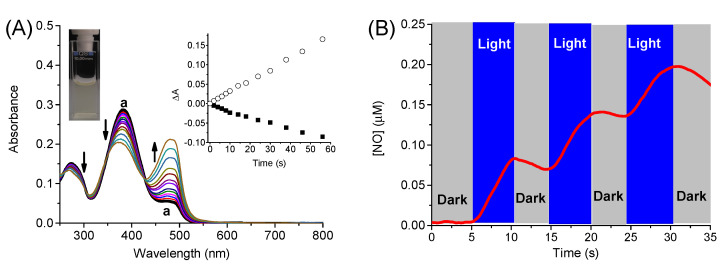
(**A**) Absorption spectrum of the supramolecular complex γ-CDng/NBF-NO (**a**) and spectral changes observed upon irradiation of this sample at λ_exc_ = 405 nm (ca. 30 mW cm^−2^). The arrows indicate the course of the spectral profile with the illumination time. The insets show the actual image of sample **a** taken under ambient light (left) and the absorbance changes at λ = 382 (■) and 480 nm (○), respectively (right). (**B**) NO release profile (λ_exc_ = 405 nm) observed for γ-CDng/NBF-NO. [γ-CDng] = 1 mg mL^−1^; [NBF-NO] = 20 µM; T = 25 °C.

Solubility studies were performed by using a concentration of γ-CDng of 1 mg mL^−1^. Thin films of NBF-NO, obtained by drying a methanol solution of the NOPD at different concentrations, were stirred with an aqueous solution of γ-CDng, and the absorption spectra of the final solutions were recorded. The absorbance values at 380 nm (absorption maximum of NBF-NO) increased as a result of the encapsulation of the NOPD within the γ-CDng to form the γ-CDng/NBF-NO host–guest supramolecular assembly. A solubility limit of 5.5 μg mL^−1^ and an encapsulation efficiency of ca. 97% were obtained. Figure 1A (spectrum **a**) shows a representative absorption spectrum of the γ-CDng/NBF-NO complex. Both the absorption maximum and the spectral shape were very similar to those previously observed in methanol/water solution [26], accounting for the absence of significant self-aggregation of the guest within the host. This was confirmed by the photoreactivity of NBF-NO observed upon blue light irradiation. The absorption spectral changes reported in Figure 1 show the bleaching of the absorption band at 380 nm, accompanied by the formation of a new, intense absorption at 485 nm (inset Figure 1A).

Apart from the small shift due to the different polarity of the environment experienced by the NOPD, the observed spectral changes are basically similar to those already observed in methanol/water solution [26] and according with the formation of NBF as stable photoproduct. NBF is, in fact, characterized by the charge transfer band typical of the amino-nitrobenzofurazan moiety, which is restored after NO release and subsequent H-abstraction from the host by the anilinyl radical intermediate [26]. According to this view, NO release was observed via a direct method using an ultrasensitive NO electrode. Figure 1B shows that instantaneous release of NO occurs from the supramolecular nanoassembly upon blue light irradiation. The NO release is exclusively regulated by light stimuli as confirmed by alternating different light/dark cycles. The quantum yield for the NO photorelease, Φ_NO_, was ~0.16, a value very similar to that observed in methanol/water solution [26]. This similarity supports well the absence of self-quenching processes between the NOPD within the nanogel. Furthermore, this high photoreactivity is also the result of the active role of the CD units of the host, which provide easily abstractable hydrogens close to the anilinyl radical intermediate generated after the NO photorelease.

**Figure 2:**
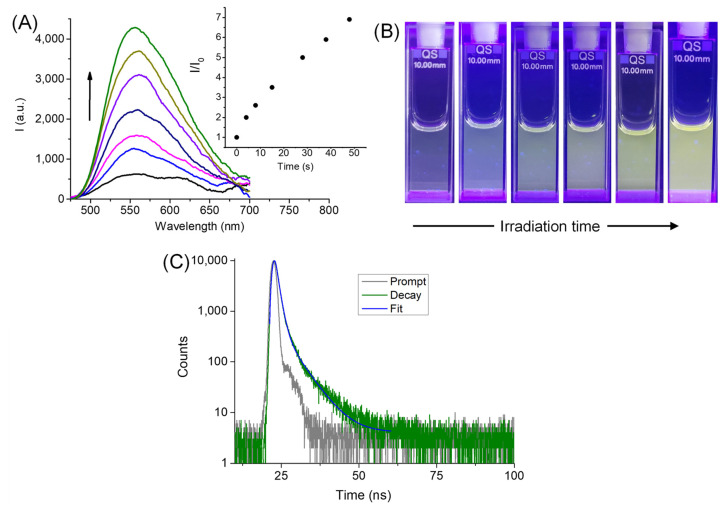
(**A**) Evolution of the fluorescence emission spectra recorded at λ_exc_ = 427 nm (isosbestic point) of the supramolecular complex γ-CDng/NBF-NO upon irradiation at λ_exc_ = 405 nm (ca. 30 mW cm^−2^). The arrow indicates the course of the spectral profile from 0 to 48 s. The inset shows variation of the fluorescence intensity monitored at λ_em_ = 550 nm; I and I_0_ represent the fluorescence intensities after each step of irradiation and the initial value before irradiation, respectively. (**B**) Actual images of the γ-CDng/NBF-NO acquired under illumination with a blue LED at different irradiation times. (**C**) Fluorescence decay profile acquired at the end of the photolysis and monitored at λ_em_ = 530 nm. [γ-CDng] = 1 mg mL^−1^; [NBF-NO] = 20 µM; T = 25 °C.

Analogously to its free form in organic solvent, NBF-NO exhibits low fluorescence emission when entrapped within the γ-CDng. However, irradiation of the supramolecular complex leads to a revival of the emission with λ_max_ = 555 nm, typical of the NBF fluorophore (Figure 2A). The appearance of the green emission of the optical reporter NBF is well visible even to naked eye (Figure 2B) and provides readable information about the photogeneration of NO. The fluorescence decay observed at the end of the photolysis (Figure 2C) shows a bi-exponential behavior with the fast and the slow components having lifetimes τ_f_ = 1.02 ns (87.08%) and τ_s_ = 4.78 ns (12.92%). This bi-exponential decay probably reflects the different host compartments experienced by the NBF fluorophore inside the γ-CDng.

**Figure 3:**
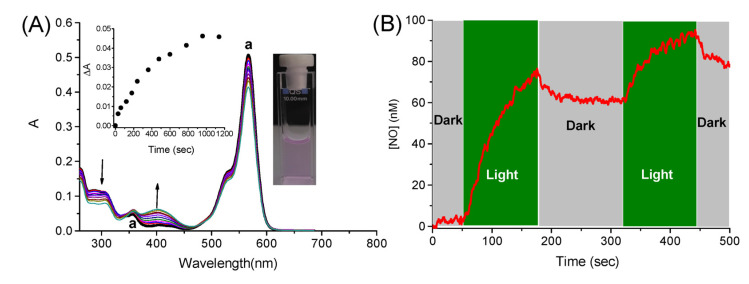
(**A**) Absorption spectrum of the supramolecular complex γ-CDng/RHD-NO (**a**) and spectral changes observed upon exposure of this sample at λ_exc_ = 532 nm (ca. 200 mW cm^−2^). The arrows indicate the course of the spectral profile with the illumination time. The insets show the actual image of sample **a** taken under ambient light (right) and the difference absorbance changes at λ = 400 nm (left). (**B**) NO release profile (λ_exc_ = 532 nm) observed for γ-CDng/NBF-NO. [γ-CDng] = 1 mg mL^−1^; [RHD-NO] = 6 µM; T = 25 °C.

RHD-NO is based on the rhodamine chromophore, a green light antenna covalently linked to a nitroso-derivative appendage through a flexible spacer [27]. The mechanism of the NO release in this NOPD involves an intramolecular photoinduced electron transfer between the excited antenna and the nitroso moiety. The anilinyl radical intermediate generated after homolytic rupture of the N-NO bond leads to the stable photoproduct RHD after H-abstraction from the solvent (Scheme 1). In contrast to NBF-NO, RHD-NO shows a persistent and intense red/orange emission which does not change significantly after NO releases. Analogously to NBF-NO, RHD-NO is insoluble in water medium. However, also in this case, this NOPD can be effectively encapsulated in the γ-CDng to form the γ-CDng/RHD-NO host–guest supramolecular complex. A solubility limit of 4.8 μg mL^−1^ and an encapsulation efficiency of ca. 55% were obtained. Figure 3A (spectrum **a**) shows a representative absorption spectrum of the γ-CDng/RHD-NO complex and the actual image of the sample which shows the typical color of the rhodamine chromophore (inset Figure 3A). The close similarity of both absorption maximum and spectral shape to those previously observed in methanol/water solution [27] accounts for the absence of relevant self-aggregation processes of the guest within the host compartment. Interestingly, the encapsulation of the NOPD within the γ-CDng does not modify its photochemical reactivity. As illustrated in Figure 3A, irradiation of the complex with green light at 532 nm induces spectral changes characterized by the formation of a new absorption at ca. 400 nm. This band arises from the push–pull character typical of the nitroaniline moiety of the stable photoproduct RHD (Scheme 1), whose formation was complete in ca. 15 min (inset Figure 3A). According to this view, NO release exclusively regulated via green light stimuli was confirmed by its direct monitoring by alternating different light/dark cycles (Figure 3B). The quantum yield for the NO photorelease, Φ_NO_, was ~10^−3^, a value very similar to that reported in methanol/water solution [27].

**Figure 4:**
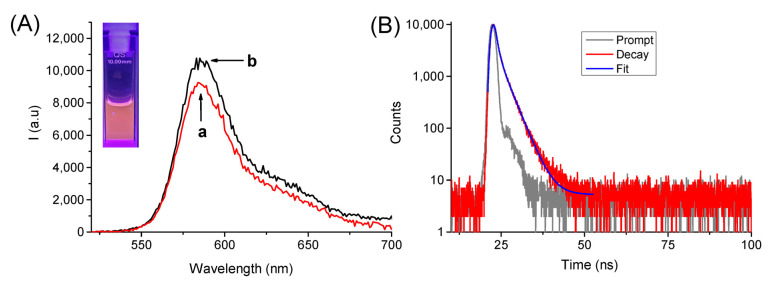
(**A**) Fluorescence emission spectra recorded at λ_exc_ = 510 nm of the supramolecular complex γ-CDng/RHD-NO before (**a**) and after (**b**) photolysis with green light. The inset shows the actual image of the γ-CDng/RHD-NO acquired under illumination with a blue LED (**B**) Fluorescence decay profile of γ-CDng/RHD-NO acquired before photolysis and monitored at λ_em_ = 587 nm. [γ-CDng] = 1 mg mL^−1^; [RHD-NO] = 6 µM; T = 25 °C.

As shown in Figure 4A (spectrum **a**), RHD-NO also exhibits a red/orange fluorescence emission typical of the rhodamine fluorophore when encapsulated within the γ-CDng, according to the photophysical behavior observed for the free guest [27]. The emission can be observed even with the naked eye (inset Figure 4A) and was well preserved after the irradiation (spectrum **b** in Figure 4A). The fluorescence decay was bi-exponential with lifetimes of τ_f_ = 0.50 ns (63.70%) and τ_s_ = 2.10 ns (36.30%), respectively. The fluorescence decay after the photolysis was complete exhibited again a bi-exponential behavior with slight changes in the lifetimes and the related amplitudes, being, τ_f_ = 0.70 ns (52.40%) and τ_s_ = 2.70 ns (47.60%), respectively. Analogously to what was already observed for NBF-NO, the bi-exponential behavior could be due to the different environments experienced by the fluorophore within the host compartment.

Preliminary antibacterial activity of the supramolecular complexes γ-CDng/NBF-NO and γ-CDng/RHD-NO and, for comparison of the only host, γ-CDng were evaluated on Gram-positive methicillin-resistant *Staphylococcus aureus* (MRSA. This bacterium is responsible for higher rate of morbidity due to its high antibiotic-resistance pattern towards traditional antibiotics. The bacterial cultures were either kept in the dark or irradiated at different times with visible light (see experimental).

The results reported in Table 1 show that the empty γ-CDng does not have any significant effect on the bacterial load either in the dark or under illumination. No significant reduction was also observed for γ-CDng/NBF-NO under the same experimental conditions. In contrast, a significant inhibitor effect was observed in the case of γ-CDng/RHD-NO under light irradiation in a fashion dependent on the irradiation time, as confirmed by the reduction of ca. 3 and more than 5 log units after 4 and 8 min of irradiation, respectively.

**Figure 5:**
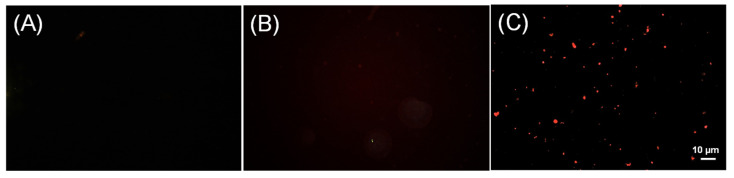
Fluorescence microscopy images obtained after incubation and following washing of (**A**) γ-CDng/NBF-NO, (**B**) γ-CDng/NBF, and (**C**) γ-CDng/RHD-NO with *S. aureus* (MRSA) ATCC 43300.

By taking into account the high NO photogeneration quantum yield of γ-CDng/NBF-NO compared to γ-CDng/RHD-NO (see above), the results obtained seem quite surprising. To gain insights into this apparent incongruence and taking advantage of the fluorescence properties of both NOPDs, we carried out fluorescence microscopy investigations. Figure 5 shows representative images obtained after incubating the bacteria with the NOPDs for 20 min, followed by washing (see experimental). No detectable green fluorescence was observed in the case of the γ-CDng/NBF-NO (poorly fluorescent) and its highly fluorescent photoproduct γ-CDng/NBF (Figure 5A,B). In contrast, clearly visible red fluorescence was observed for γ-CDng/RHD-NO. These findings suggest that the difference in the antibacterial action of the two NOPDs is probably due to their different interaction with bacteria.

## 3. Materials and Methods

### 3.1. Materials

All chemicals were purchased from Sigma-Aldrich and used without further purification. γ-CDng, γ-CDng/NBF-NO and γ-CDng/RHD-NO were prepared according to our previously reported procedures [25,26,27]. All solvents used for the spectrophotometric studies were of spectrophotometric grade. MilliQ water was used for polymer solubilization and all chemical and photochemical experiments.

### 3.2. Instrumentation

UV-Vis absorption spectra were recorded with a JascoV-560 spectrophotometer. Vis fluorescence emission spectra were recorded with a Spex Fluorolog-2 (mod. F-111) spectrofluorimeter. All spectra have been collected under air-equilibrated conditions using quartz cells with a path length of 1 cm. Fluorescence decays were acquired with the same fluorimeter as above equipped with a TCSPC Triple Illuminator. The samples were excited with a pulsed diode excitation source (Nanoled) at λ_exc_ = 455 nm, and the decays were collected at λ_em_ = 530 and 587 nm for γ-CDng/NBF-NO and γ-CDng/RHD-NO, respectively. The system allowed measurement of fluorescence lifetimes with a time resolution of 200 ps. The multiexponential fit of the fluorescence decay was obtained using the following equation:

I(t) = Σα_i_exp(^−t/τi^)

where I is the fluorescence intensity, α is the relative amplitude and τ is the lifetime. 

All photolysis experiments were performed by irradiating the samples in thermostated quartz cells (with path length of 1 cm and capacity of 3 mL) under gentle stirring, using a continuum laser at λ_exc_ = 405 nm or λ_exc_ = 532 nm and having a beam diameter of ca. 1.5 mm.

NO release in solution was monitored by a direct method through amperometric detection with an ISO-NO meter (World Precision Instruments) having short response time (<5 s) and sensitivity in the range 1 nM–20 µM and equipped with a data acquisition system. The analogue signal was digitalized with a four-channel recording system and then transferred to a PC. The NO sensor was firstly accurately calibrated by known amounts of NO produced by mixing standard solutions of NaNO_2_ with 0.1 M H_2_SO_4_ and 0.1 M KI according to the following reaction:

4H^+^ + 2I^−^ + 2NO_2_^−^ → 2H_2_O + 2NO + I_2_

Irradiation was performed in a thermostated quartz cell (1 cm path length, 3 mL capacity) using the continuum laser at λ_exc_ = 405 nm or λ_exc_ = 532 nm. To avoid NO signal artefacts due to photoelectric interference on the ISO-NO electrode, all NO measurements were carried out under gentle stirring, positioning the electrode outside the light path.

The size distribution was determined on a Zetasizer Nano ZS (Malvern Instruments Ltd., Malvern, UK).

### 3.3. NO Photorelease Quantum Yields

NO photorelease quantum yields, Φ_NO,_ were determined within the 20% transformation of γ-CDng/NBF-NO and γ-CDng/RHD-NO by using the following equation:

Φ_NO_ = [**T**] × V/t × (1 − 10^−A^) × I

where [**T**] is the concentration of phototransformed γ-CDng/NBF-NO and γ-CDng/RHD-NO, V is the volume of the irradiated sample, t is the irradiation time, A is the absorbance of the sample at the excitation wavelength and I the intensity of the excitation light source. The concentration of the phototransformed γ-CDng/NBF-NO and γ-CDng/RHD-NO were determined spectrophotometrically by taking into account the absorption changes at λ = 382 nm (∆ε_382_ = 12,000 M^−1^ cm^−1^) and λ = 400 nm (∆ε_400_ = 7500 M^−1^ cm^−1^). I was calculated via potassium ferrioxalate actinometry.

### 3.4. Preparation of the Supramolecular Complexes

Stock solutions of NBF-NO and RHD-NO were prepared in MeOH. Afterwards the solvent was evaporated under reduced pressure at 25 °C. The resulting film was rehydrated with an aqueous solution of γ-CDng (1 mg mL^−1^) by stirring for 4 h at room temperature.

Encapsulation efficiency (EE %) was calculated using the following:

EE % = (W_IN_/W_i_) × 100

where W_IN_ is the amount of drug in the nanoassembly and Wi is the total amount of drug added initially during preparation.

### 3.5. Biological Assays

#### 3.5.1. Antibacterial Activity

The activity of the different samples was investigated against *Staphylococcus aureus* (MRSA) ATCC 43300. The overnight broth cultures grown in Mueller Hinton Broth (MHB) were centrifuged at 3500 rpm for 15 min, washed with phosphate-buffered saline (PBS) at pH 7.2 and resuspended in order to obtain a final density of approximatively 1–2 × 10^7^ colony forming unit (CFU) mL^−1^ by optical density measurements at 570 nm. Twenty µL of the bacterial suspension were placed into a 96-well microplate containing 180 µL of each Hydrogel sample. The microplate was irradiated for 4′ and 8′ by using a 150 W Xe lamp equipped with a cut-off filter at λ > 400 nm or kept in the dark. At the end of the irradiation each sample whole and serially diluted in PBS was drop-plated in duplicate on Mueller Hinton Agar (MHA) plate and incubated at 37 °C for 24–48 h. Later, colonies were counted at the most appropriate dilution and expressed as CFU mL^−1^. PBS was included as growth control.

#### 3.5.2. Fluorescence Microscopy

The overnight broth culture of methicillin-resistant *S. aureus* (MRSA) ATCC 43300 was washed twice and suspended in PBS. The cell suspension (10^8^ CFU mL^−1^) was treated with different samples and incubated under dark conditions for 20 min. Controls without samples were included. After incubation, the bacterial cells were harvested by centrifugation at 12,000 rpm for 10 min and washed twice with PBS. The cell suspensions were spotted onto glass slides and then the coverslips were mounted. The images were analyzed using a LEICA fluorescence microscope (Leitz, Wetzlar, Germany).

## 4. Conclusions

We have shown that two water-insoluble NOPDs can be supramolecularly encapsulated within a water dispersible γ-CDng with excellent preservation of their photochemical properties. Excitation of the γ-CDng/NBF-NO and γ-CDng/RHD-NO nanoassemblies with blue and green light, respectively, triggers the NO release with quantum efficiencies very similar to those observed for the free gusts in the organic solvent. Moreover, the photoactivatable and persistent fluorescence properties of NBF-NO and RHD-NO, respectively, are well maintained in the supramolecular complexes, with only minor changes observed in the fluorescence lifetimes. We outline that such results are not trivial. In fact, in many cases, the photobehavior of guest molecules confined in a restricted host nanoenvironment in the presence of steric constrains and specific interactions may lead to significant modification of the main photodeactivation pathways observed for the free guests in the nature, efficiency or both. In the present case, the preservation of nature and efficiency of the photochemical processes is mainly the result of (i) the lack of self-aggregation/quenching processes of the NOPDs, although confined in a small-sized host, in which they are preferentially encapsulated as monomeric forms; (ii) the key role that the CD units of the nanogel play as good hydrogen donors towards the nitrogen-centered radical intermediates generated after the light-triggered homolytic rupture of the N-NO bond.

Preliminary antibacterial tests carried out on MRSA have demonstrated that γ-CDng/RHD-NO is very active in inhibiting the bacteria load, although this NOPD produces NO with efficiency lower than that of γ-CDng/NBF-NO, which, in contrast, shows only negligible antibacterial activity. Fluorescence microscopy experiments suggest that this apparent discrepancy could be due to the different affinity of the NOPDs for the bacterial cells.

## Figures and Tables

**Table 1:** Activity of different samples against methicillin-resistant *S. aureus* (MRSA) ATCC 43300 in the dark and under visible light irradiation. Results are reported as the mean of three separate measurements on three different batches ± SD.

Sample	Time (min)	Dark Log CFU mL^−1^	Light Log CFU mL^−1^
Inoculum	0	6.36 ± 0.03	
PBS	4	6.23 ± 0.03	6.19 ± 0.06
γ-CDng	4	6.18 ± 0.10	6.15 ± 0.04
γ-CDng/NBF-NO	4	6.07 ± 0.08	6.07 ± 0.04
γ-CDng/RHD-NO	4	6.15 ± 0.11	3.36 ± 0.08
PBS	8	6.21 ± 0.08	6.14 ± 0.09
γ-CDng	8	6.17 ± 0.05	6.13 ± 0.05
γ-CDng/NBF-NO	8	6.07 ± 0.09	6.06 ± 0.09
γ-CDng/RHD-NO	8	5.84 ± 0.04	1.46 ± 0.15

## Data Availability

Not applicable.

## Sample Availability

Not available.
